# *KIT* Mutation-*NTRK* fusion oncogenic driver switch: a novel mechanism of acquired imatinib resistance in GIST

**DOI:** 10.1038/s41698-026-01289-1

**Published:** 2026-01-21

**Authors:** Simona Gloazzo, Marta Sbaraglia, Elena Bellan, Daniela Gasparotto, Elena Belli, Davide Baldazzi, Gabriella Rossi, Elena Magnani, Maria Pia Rosito, Andrea Carnevali, Sara Piccinin, Angelo Paolo Dei Tos, Roberta Maestro

**Affiliations:** 1 Unit of Oncogenetics and Functional Oncogenomics, Centro di Riferimento Oncologico di Aviano (CRO Aviano) IRCCS, National Cancer Institute, Aviano, Italy; 2 Department of Integrated Diagnostics, Azienda Ospedale-Università Padova, Padova, Italy; 3 Department of Medicine, University of Padua School of Medicine, Padova, Italy; 4 Oncology Department, San Donato Hospital, USL Sud Est, Toscana Arezzo, Italy; 5 Oncology Department, Ospedale di Sansepolcro, USL Sud Est, Toscana Sansepolcro, Italy; 6 Department of Surgical Pathology, San Donato Hospital, USL Sud Est, Toscana Arezzo, Italy

**Keywords:** Cancer, Oncology

## Abstract

Imatinib is the first-line treatment for advanced gastrointestinal stromal tumors (GISTs) harboring *KIT* or *PDGFRA* mutations. Unfortunately, resistance invariably develops, typically through secondary *KIT*/*PDGFRA* mutations. Here, we describe an unprecedented case of acquired imatinib resistance associated with an oncogenic driver switch, from a *KIT* mutation to an *NTRK3* fusion. The index case was a *KIT* exon 11-mutated gastric GIST that progressed on imatinib. Despite retaining the original *KIT* mutation and DOG1 expression, the relapsed tumor lost KIT expression and exhibited a dedifferentiated phenotype. Transcriptomic profiling revealed a *de novo EML4::NTRK3* gene fusion. In vitro modeling demonstrated that *EML4::NTRK3* confers imatinib resistance, while sensitizing GIST cells to NTRK inhibitors. This first reported instance of an *NTRK* fusion as a secondary event in GIST progression underscores the importance of testing for *NTRK* alterations in tumors that have developed resistance to tyrosine kinase inhibitors to ensure patients are offered all available therapeutic options.

## Introduction

Gastrointestinal stromal tumors (GISTs) are the most common mesenchymal neoplasms of the gastrointestinal tract. The majority of GISTs are driven by activating mutations in *KIT* or *PDGFRA*, while less common drivers include mutations in *BRAF*, *NF1*, or alterations in the *SDH* complex. More recently, a small subset of GISTs has been found to harbour oncogenic *NTRK* fusions^1^. Like KIT and PDGFRA, NTRK is a tyrosine kinase receptor that promotes cell proliferation and survival through MAPK/ERK, PI3K/AKT, and JAK/STAT signaling pathways. Therefore, both oncogenic *KIT* mutations and *NTRK* fusions lead to ligand-independent constitutive activation of convergent downstream signaling cascades^2–4^.

Tyrosine kinase inhibitors (TKIs) are the standard of care for advanced *KIT/PDGFRA* mutant GISTs, with imatinib as the first-line agent^1,5–7^. However, secondary resistance to these treatments eventually develops, often due to additional on-target gene mutations that impair drug binding^8–17^ or, less frequently, off-target gene alterations^18–20^ (Table 1).

**Table 1:** Mechanisms of acquired imatinib resistance identified in GIST patients with primary *KIT* or *PDGFRA* imatinib-sensitizing mutations

On-target resistance mechanisms	
	**Median frequency (%)** ***(min-max)***
*KIT* mutations in the ATP-binding pocket (exons 13–14)^8–17^	30.6 *(14.3–64.3)*
*KIT* mutations in the activation loop (exons 17–18)^8–17^	31.7 *(14.3–72.7)*
*KIT* gene amplification^9,10,15^	< 10
*PDGFRA* mutations in the activation loop (exon 18)^10,14,16^	2.2 *(1.7–3.2)*
**Rarer off-target resistance mechanisms**	
*AXL* overexpression^18^, *BRAF* V600E mutation^19^, *FGFR2::TACC2* fusion^20^, *EML4::NTRK3* fusion^this study^	

Here, we report a case of acquired imatinib resistance associated with an oncogenic driver switch, from *KIT* exon 11 mutation to *EML4::NTRK3* fusion. To our knowledge, this is the first report of an *NTRK3* fusion being involved as a mechanism of oncogenic driver switch and imatinib resistance in GISTs. Since *NTRK* fusion-positive tumors have been shown to be sensitive to NTRK inhibitors^21,22^, the case presented here discloses new therapeutic opportunities for the treatment of GISTs that progress under TKIs therapy.

## Results

### Clinical case

The index case was a 35-years old man who was admitted to the Arezzo General Hospital in November 2014 for abdominal pain. Computed tomography (CT) showed a large gastric mass and several diaphragm nodules. The patient underwent partial gastrectomy and splenectomy.

**Fig. 1:**
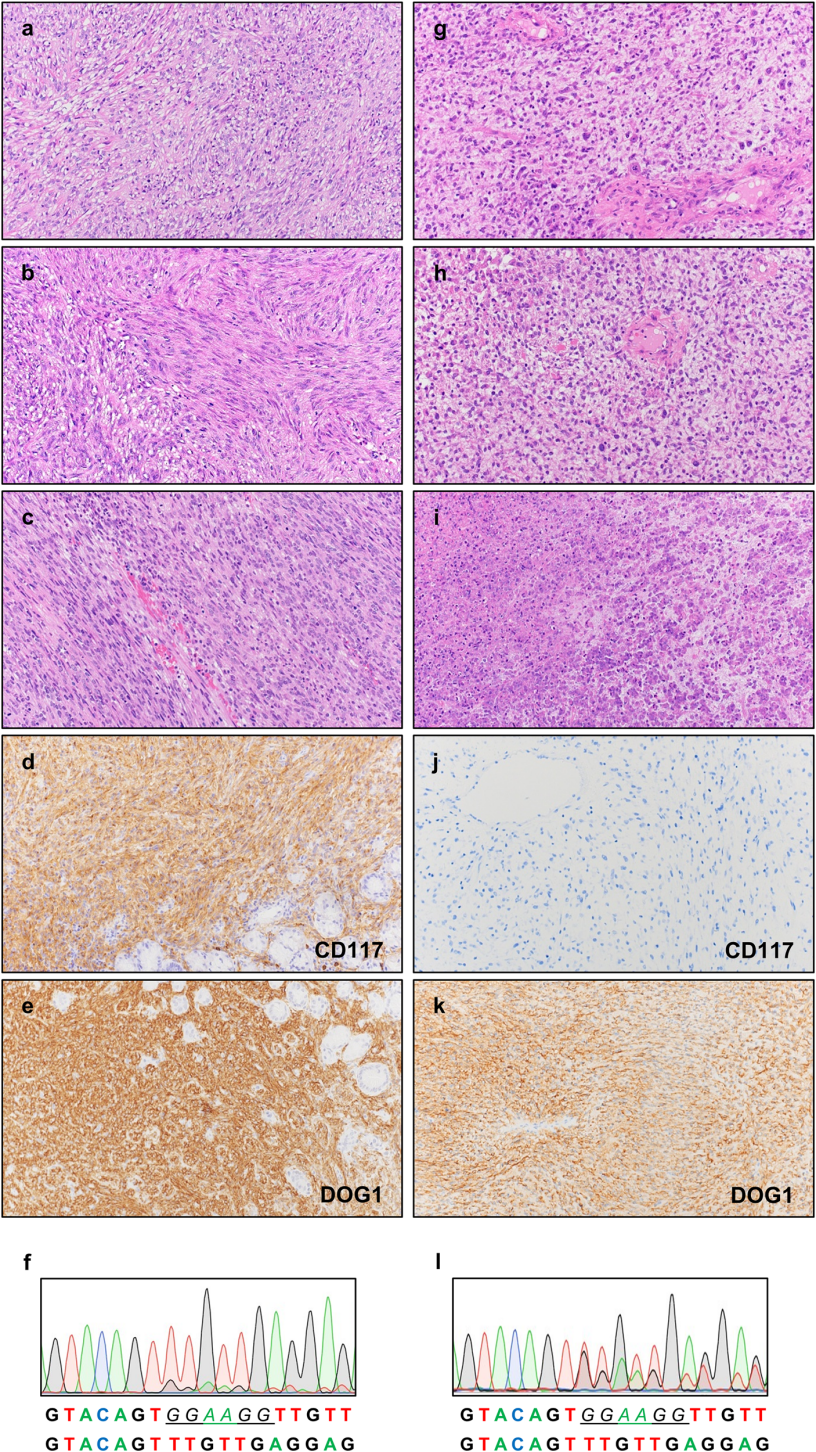
Morphological and immunophenotypic features and *KIT* mutation status of the primary GIST (left) and the corresponding relapse (right). **a**, **b** Primary resection specimen showing spindle cell morphology with paranuclear vacuolation admixed with areas of increased cellularity and nuclear atypia. **c** Numerous mitotic figures were detected. **d**, **e** Tumor cells display strong, diffuse expression of CD117 and DOG1. **f** DNA mutation analysis of the primary GIST (tumor cell content 90%) revealed the presence of an imatinib-sensitizing *KIT* exon 11 mutation consisting of a 6-nucleotide deletion (c.1670_1675del, corresponding to pW557_V559delinsF), underlined in the figure. **g**–**i** Tumor relapse exhibits atypical undifferentiated spindle and ovoid cells within a myxoid stroma, with areas of necrosis and a high mitotic index, including atypical mitoses. **j**, **k** The neoplastic cells are negative for CD117, while strong multifocal expression of DOG1 was maintained. **l** The same pW557_V559delinsF *KIT* mutation detected in the primary tumor was present in the DNA of the recurrence (tumor cell content 60%). Images were taken at 10X magnification.

Histological analysis revealed that the tumor was composed of monomorphic spindle cells with scant eosinophilic cytoplasm and paranuclear vacuolation arranged in a fascicular pattern of growth (Fig. 1a, b). Numerous non-atypical mitotic figures were detected (approximately 50 mitoses/5 mm^2^) (Fig. 1c). Tumor cells showed strong and diffuse expression of CD117 and DOG1 (Fig. 1d, e). Molecular analysis identified an imatinib-sensitizing *KIT* exon 11 mutation (c.1670_1675del, corresponding to pW557_V559delinsF) (Fig. 1f). The final diagnosis was of metastatic GIST with conventional morphology.

**Fig. 2:**
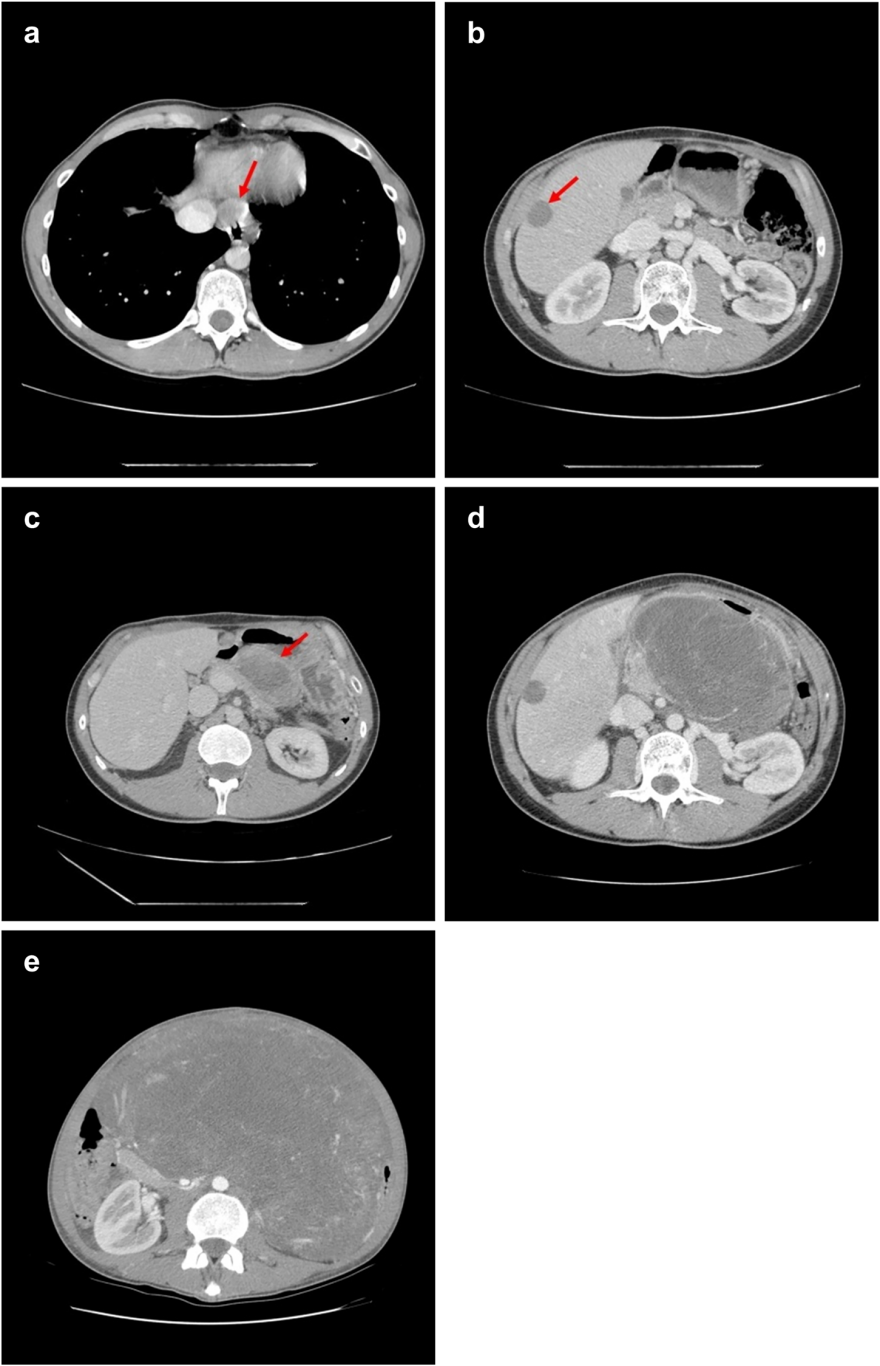
CT imaging of the patient under treatment. **a**, **b** Follow-up CT scan from September 2017 shows a partial response to imatinib, with reduced size of periesophageal and hepatic lesions (red arrows). **c** By February 2018, CT imaging reveals disease progression, with new intra-abdominal lesions, including an 8 cm perigastric mass with central necrosis (red arrow). **d** Following imatinib dose escalation, further progression is observed, including enlargement of the perigastric mass. **e** Imaging obtained at the late-stage of disease showing a large tumor mass occupying most of the abdominal cavity.

Post-surgical imaging confirmed disease-free status. Considering the advanced status of the disease, adjuvant imatinib (400 mg/day) was initiated (February 2015) but discontinued after 18 months at the patient’s request. Clinical follow-up was maintained thereafter. In June 2017 (31 months post-diagnosis), the patient presented with epigastric pain and a palpable mass; imaging revealed a 26 mm hepatic lesion and multiple epigastric nodules consistent with neoplastic recurrence. Imatinib (400 mg/day) was restarted, leading to marked regression of lesions (Fig. 2a, b). In February 2018 (39 months post-diagnosis), PET/CT (Positron Emission Tomography/Computed Tomography) showed diffuse peritoneal “sarcomatosis”, including a gastric mass, and peritoneal and periesophageal nodules (Fig. 2c). Imatinib was escalated (800 mg/day) but disease progressed (Fig. 2d). Debulking surgery was performed (April 2018).

Pathological examination revealed an atypical undifferentiated spindle and ovoid cell morphology set in a myxoid stroma. Diffuse areas of necrosis were present and mitotic index was elevated, including atypical forms (Fig. 1g–i). The tumor was CD117 negative but showed strong, albeit multifocal, DOG1 positivity (Fig. 1j, k). Molecular analysis of tumor DNA revealed the *KIT* exon 11 mutation (pW557_V559delinsF) originally detected in the primary lesion (Fig. 1l). No canonical *KIT* resistance mutation was identified. The combination of clinical history, immunophenotype, and molecular data, despite the absence of classic GIST morphology, was consistent with the diagnosis of relapse of GIST that had undergone dedifferentiation. Sunitinib (37.5 mg/day) was initiated but the patient experienced rapid disease progression and died shortly after (June 2018) (Fig. 2e).

### RNA-sequencing identifies a novel *EML4::NTRK3* fusion transcript in the relapse

**Fig. 3:**
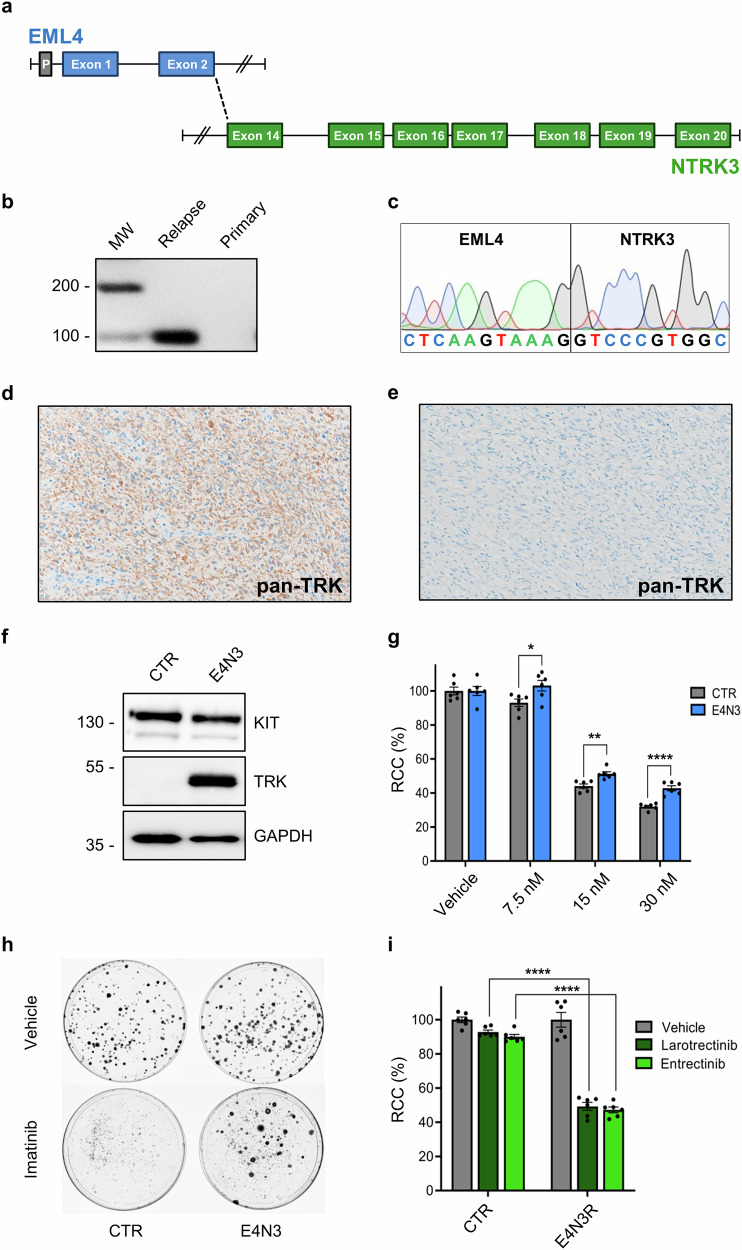
Identification of the *EML4::NTRK3* fusion and in vitro cell modeling. **a** Schematic representation of the *EML4::NTRK3* fusion identified by RNA-sequencing in the relapse. **b** The selective expression of the *EML4::NTRK3* fusion transcript in the relapse but not in the primary tumor was confirmed by RT-PCR. **c** Sequencing of the RT-PCR product of the relapse displaying the junction between *EML4* and *NTRK3*. **d** Immunohistochemistry for pan-TRK shows strong and diffuse staining in the recurrence. **e** Negative pan-TRK immunostaining in the primary tumor (10X magnification). **f** Western blot analysis for KIT and the NTRK-fused protein product in the GIST-E4N3 cell model engineered to express the *EML4::NTRK3* chimera. GIST-CTR cells served as a negative control. GAPDH was used as a loading control. A representative image of three biologically independent replicates is shown. **g** GIST-E4N3 cells show reduced sensitivity to imatinib relative to GIST-CTR. Relative cell count (RCC) was assessed by the SRB assay at 48 h post-treatment. *p-value ≤ 0.05, **p-value ≤ 0.01, ****p-value ≤ 0.0001. Data are presented as mean ± SEM (*n* = 6). **h** Colony growth assay demonstrating the capacity of GIST-E4N3 cells to survive a chronic, 3-week imatinib treatment (50 nM). For better visualization of individual colonies of vehicle-treated cells, images taken at 2-week DMSO treatment are shown. **i** Compared to GIST-CTR, imatinib-resistant derivatives of GIST-E4N3 cells (GIST-E4N3R) exhibit increased sensitivity to NTRK inhibitors (200 nM larotrectinib, dark green; 200 nM entrectinib, light green; 48 h-treatment). ****p-value ≤ 0.0001. Data are presented as mean ± SEM (*n* = 6).

To gain insights into the mechanisms of imatinib resistance and GIST dedifferentiation, both primary tumor and relapse were subsequently transcriptionally profiled by RNA-sequencing. In line with immunohistochemistry, the relapse showed a dramatic decrease in *KIT* mRNA levels. Interestingly, RNA-sequencing revealed an in-frame *EML4::NTRK3* fusion, which involves exons 1–2 of *EML4* (chr 2p21, NM_019063) and exons 14–20 of *NTRK3* (chr 15q25.3, NM_001012338). EML4 (Echinoderm Microtubule Associated Protein Like 4) is a microtubule-associated protein that has been reported to participate in oncogenic gene fusion events in various cancers^23,24^. NTRK3 (Neurotrophic Receptor Tyrosine Kinase 3) is a member of the tropomyosin receptor kinase family that, upon binding its ligand NTF3 (neurotrophin-3), undergoes autophosphorylation and activates pro-proliferative signaling cascades, including MAPK/ERK, PI3K/AKT, and JAK/STAT^4^. In the identified *EML4::NTRK3* fusion, the 429-amino acid chimeric protein encompasses the EML4 coiled-coil domain (aa 1–69) and the NTRK3 kinase domain (aa 70–429) (Fig. 3a; Supplementary Table 1). No in-frame fusion was detected in the primary tumor. RT-PCR confirmed the expression of the *EML4::NTRK3* chimera solely in the relapse, and Sanger sequencing validated the breakpoint identified by RNA-sequencing (Fig. 3b, c; Supplementary Fig. S1). The *EML4* gene is well expressed in GISTs (data not shown), indicating that the *EML4* promoter likely supports significant expression of the fusion. Accordingly, immunohistochemistry for pan-TRK showed strong and diffused staining in the relapse whilst the primary tumor was negative (Fig. 3d, e).

Overall, these data indicate that after imatinib treatment the tumor acquired an *EML4::NTRK3* gene fusion while losing the expression of the activated *KIT* allele. This suggests that an oncogenic driver switch occurred during treatment, and that the expression of the EML4::NTRK3 fusion product supports imatinib resistance.

### *EML4::NTRK3*-expressing cells are resistant to imatinib and sensitive to NTRK inhibitors

To investigate the hypothesis that the EML4::NTRK3 fusion product supports the proliferation of GIST cells under imatinib pressure, we utilized the GIST-T1 cell line^25^. This cell line carries an imatinib-sensitizing, heterozygous *KIT* exon 11 mutation (p.Val560_Tyr578del), thereby mimicking the genetic context of the primary tumor in the index case. GIST-T1 cells were engineered to ectopically express the *EML4::NTRK3* chimera (GIST-E4N3), while cells transduced with the empty vector served as a control (GIST-CTR) (Fig. 3f; Supplementary Fig. S2).

Compared to GIST-CTR cells, GIST-E4N3 cells exhibited reduced sensitivity to imatinib under both acute (Fig. 3g) and chronic (Fig. 3h) treatment conditions. Specifically, in both the SRB cell viability assay performed after a 48-hour treatment with imatinib at different dosages, and the colony growth assay evaluated after 3 weeks of treatment, GIST-E4N3 cells showed increased survival. These results suggest that the *EML4::NTRK3* fusion transcript confers a growth advantage to GIST-T1 cells under imatinib pressure.

Imatinib-resistant GIST-E4N3 cells (GIST-E4N3R), obtained following prolonged imatinib challenge, were evaluated for their response to NTRK inhibitors. Compared to GIST-CTR cells, GIST-E4N3R cells exhibited increased sensitivity to both larotrectinib and entrectinib, supporting the relevance of the *EML4::NTRK3* fusion in their viability (Fig. 3i).

## Discussion

Although systemic treatment with imatinib or other TKIs can achieve long-term disease control in patients with *KIT* or *PDGFRA* mutations, most GISTs eventually progress due to the development of acquired resistance. Imatinib resistance is commonly associated with the acquisition of secondary mutations in the ATP-binding pocket or the activation loop of the primary driver (*KIT* or *PDGFRA*)^1,8–17^, although other mechanisms (e.g., *KIT* or *PDGFRA* amplification)^9,10,15^ or off-target gene alterations (e.g., mutations in downstream effectors or activation of alternative receptor tyrosine kinases)^18–20^ may also be involved. Recently, a *FGFR2::TACC2* gene fusion was detected in a multi TKI-resistant *KIT* exon 11-driven GIST, prompting the authors to speculate a role for the fusion in drug resistance^20^.

We and others have reported the detection of *NTRK* activating gene fusions in GISTs devoid of canonical oncogenic drivers^21,22,26^. Although the existence of this group of GISTs has been long questioned^27^, a recent survey has provided evidence that GISTs carrying *NTRK* fusions do exist and may be sensitive to NTRK inhibitors^22^. Importantly, in these tumors the fusion represents the primary driver event.

The case reported here highlights an unprecedented scenario: *NTRK* fusions can represent a mechanism of oncogenic driver switch and acquired resistance to imatinib. The oncogenic driver switch, from a *KIT* mutation to an *NTRK3* fusion, occurred after imatinib treatment and was associated with concomitant transcriptional silencing of the original *KIT* driver mutation. Notably, this molecular switch was accompanied by a morphological transition toward a dedifferentiated GIST phenotype. GIST dedifferentiation is characterized by atypical morphology, loss of canonical markers such as CD117, DOG1, and CD34, and it is often associated with long-term imatinib therapy and disease progression^28–31^. Dedifferentiation poses significant diagnostic challenges, as the dedifferentiated component may histologically mimic high-grade sarcomas or other high-grade malignancies, potentially resulting in misclassification. In our case, although the post-imatinib tumor exhibited atypical morphology, the maintenance of the original *KIT* exon 11 mutation at the genomic level and the expression of DOG1 supported the diagnosis of dedifferentiated GIST. This highlights the importance of integrating immunohistochemistry, molecular, and clinical data to ensure an accurate diagnosis and avoid diagnostic pitfalls.

To date, approximately 20 cases of GISTs harboring *NTRK* fusions have been reported in the literature, with CD117 and/or DOG1 expression documented in about half of them^22^. Our study adds further support to the notion that *NTRK* fusion-driven GISTs do exist and represent a distinct molecular entity. Importantly, to the best of our knowledge, this is the first report demonstrating an *NTRK* fusion as a mechanism of oncogenic driver switch and acquired resistance to imatinib.

Through in vitro modeling, we demonstrated that the *EML4::NTRK3* fusion confers a growth advantage under imatinib treatment to *KIT*-driven GIST cells, supporting its functional role in resistance. Moreover, the expression of the fusion conveys sensitivity to NTRK inhibitors to imatinib-resistant cells. Unfortunately, the patient did not benefit from our findings since the *EML4::NTRK3* fusion was identified through RNA sequencing after the patient had already passed away. Consequently, NTRK inhibitor therapy was not administered, and its therapeutic efficacy in our patient remains unassessed, representing a limitation of our study. It is also important to note that transcriptional profiling is not part of the standard diagnostic workflow for GIST. Therefore, our finding that *NTRK* fusions can emerge as secondary events in GIST progression underscores the clinical relevance of including *NTRK* fusion analysis into the molecular workup of GISTs with acquired TKI resistance to inform personalized treatment approaches.

## Methods

### Histopathological diagnosis

The case was sent for consultation to APDT and the revised diagnosis was made by APDT and MS based on morphology, immunophenotype, and molecular data.

Immunohistochemistry was performed on 3 μm formalin-fixed, paraffin-embedded tissue sections using a panel of antibodies to evaluate tumor phenotype. The antibodies included CD117 (polyclonal serum, dilution 1:400; Dako; Carpentaria, CA), DOG1 (clone SP31, prediluted; Cell Marque; Rocklin, CA), S100 (clone EP32, prediluted; Leica Biosystem; Nussloch, DE), SMA (clone 1A4, dilution 1:100; Cell Marque; Rocklin, CA), Desmin (clone D33, dilution 1:400; Dako; Carpentaria, CA), and pan-TRK (clone EPR17341, prediluted; Ventana; Cupertino, CA). Detection was carried out using a standard immunoperoxidase method, diaminobenzidine chromogen and hematoxylin counterstaining. The study was approved by the local ethics committee (Marca 456/CE). Informed consent was provided by the study participants. All procedures were carried out in accordance with the principles of the Declaration of Helsinki.

### DNA mutation analysis and RNA-sequencing

DNA and RNA extraction were essentially as described in ref. ^32^. Briefly, DNA and RNA were extracted from FFPE tissue sections mounted on glass slides. Areas with high tumor cell content (90% in the primary tumor and 60% in the relapse) were marked by the pathologists on a reference hematoxylin-eosin-stained slide. Corresponding regions were scraped from matched unstained slides and dewaxed with the Qiagen deparaffinization solution (Qiagen, Germantown, MD, USA). The Ambion RecoverAll™ Total Nucleic Acid Isolation Kit for FFPE (Thermo Fisher Scientific, Carlsbad, CA, USA) was used for DNA and RNA purification. *KIT*, *PDGFRA*, *SDHA-D*, *BRAF*, *H/K/NRAS* and *NF1* gene mutation analysis was performed using a targeted NGS sequencing panel^32^. For whole RNA-sequencing analysis, libraries were prepared using the Illumina TruSeq Stranded Total RNA Kit with Ribo-Zero (Illumina, San Diego, CA, USA) and processed as described^33^. Libraries were sequenced on an Illumina NextSeq550 apparatus to an average of 60 million paired-end reads per sample. The quality of the row sequencing data was evaluated with the FastQC software. Reads were aligned to the hg38 reference genome (GRCh38.p13) with STAR v2.7.3.a, and RSEM v1.3.1 was utilized for transcript quantification^34,35^. Arriba and Dragen tools were used for fusion transcript identification^36,37^.

### PCR/Sanger sequencing validation of *KIT* exon 11 mutation and *EML4::NTRK3* fusion

*KIT* exon 11 was PCR-amplified from genomic DNA of the primary tumor and relapse samples using the GoTaq® Master Mix (Promega, Madison, WI, USA) and the following primers: forward 5’-TCCAGAGTGCTCTAATGACTGAG-3’ and reverse 5’-GCCTGTTTCTGGGAAACTCC-3’. PCR-Sanger sequencing was then performed using the same primers to confirm the presence of the c.1670_1675del mutation.

Total RNA (500 ng), isolated from the primary tumor and relapse samples, was reverse-transcribed using SuperScript III (Thermo Fisher Scientific, Carlsbad, CA, USA). To confirm the expression of the *EML4::NTRK3* fusion transcript, RT-PCR was performed using the following primers: EML4 forward 5’-TTGAGGCGTCTTGCAATCT-3’ and NTRK3 reverse 5’-GCTGAGTCCTCCTCACCACTGATG-3’. The resulting PCR product was subsequently sequenced using the same primers.

### Cell model generation

LinxA were obtained from Greg Hannon (Cancer Research UK Cambridge Institute, Cambridge, UK). GIST-T1 were obtained from Cosmo Bio (Carlsbad, CA, USA). Cells were regularly authenticated by STR profiling (Supplementary Table 2). Both cell lines were maintained in DMEM (Thermo Fisher Scientific, Carlsbad, CA, USA) supplemented with 10% heat-inactivated fetal bovine serum (FBS, Thermo Fisher Scientific) and gentamicin (8 μg/mL), at 37 °C and 5% CO_2_.

To generate the GIST-E4N3 cell model, the *EML4::NTRK3* cDNA was PCR-amplified and cloned into the pLPC-puro retroviral vector as previously described^26^. LinxA cells were transfected with either the pLPC-E4N3 or the empty pLPC vector. Forty-eight hours post-transfection, the supernatants were collected, filtered through a 0.22 µm filter, and used to infect GIST-T1 cells. Infected cells were enriched by puromycin selection.

Imatinib-resistant GIST-E4N3R cells were derived by chronic imatinib treatment (20 nM for >30 days) of GIST-E4N3 cells.

### Cell viability and colony growth assays

For drug sensitivity assays, cells were seeded in sextuplicate in 96-well plates and treated the following day with imatinib, larotrectinib, or entrectinib, as indicated (Selleckchem, Munich, DE). Stock solutions of each drug were prepared at a 1000× concentration in DMSO (vehicle).

The sulforhodamine B (SRB) cell viability assay was used to evaluate cell viability after a short-term drug treatment (48 h). The relative cell count (RCC) was calculated as the percentage of cells surviving the 48h-treatment, normalized to DMSO-treated cells.

To evaluate cell survival under chronic treatment conditions, a colony growth assay was performed. GIST-E4N3 and GIST-CTR cells were seeded onto 100 mm dishes at a density of 1 × 10^4^ cells per dish. Cells were cultured for 10 days before treatment with 50 nM imatinib (Selleckchem) or vehicle (DMSO) for three weeks. Media supplemented with imatinib or DMSO was refreshed every 3–4 days. Colony growth was assessed by crystal violet staining according to a standard protocol^38^. Experiments were conducted at least in triplicate and results were confirmed on two independent cell infections. *p*-values were calculated with 2-tailed unpaired *t t*est. Data were analyzed using GraphPad Prism Software.

### Western blotting

Protein expression was determined by Western blot. Briefly, cells were lysed in RIPA buffer (Santa Cruz Biotechnology, Dallas, TX, USA). Whole cell lysates were quantified using the Bradford protein assay (Bio-Rad Laboratories, Hercules, CA, USA). Proteins were separated by SDS-PAGE on 4–15% Criterion TGX precast gels (Bio-Rad Laboratories) and transferred onto a nitrocellulose membrane (Protran Whatman, Merck, Darmstadt, DE). Proteins were detected by chemiluminescence (LiteUp kit, Euroclone, Pero, Milan, Italy). Immunoblots were scanned using a ChemiDoc MP apparatus and analyzed with the ImageLab software (Bio-Rad Laboratories). The following antibodies were used: anti-KIT (clone E-1; Santa Cruz Biotechnology) and anti-TRK (polyclonal serum; Santa Cruz Biotechnology). Anti-GAPDH (clone 6C5; Santa Cruz Biotechnology) was used for loading control.

## Supplementary information

Supplementary Table

## Data Availability

Data is provided within the manuscript or in the supplementary information file. Raw data (FASTQ files) are available on NCBI's SRA database (PRJNA1373022).

## References

[1] Cicala, C. M., Bauer, S., Heinrich, M. C. & Serrano, C. Gastrointestinal Stromal Tumor: Current Approaches and Future Directions in the Treatment of Advanced Disease. *Hematol. Oncol. Clin. North Am.* **39**, 773–784 (2025). 40368739 10.1016/j.hoc.2025.04.006 PMC12416134

[2] Szucs, Z. et al. Molecular subtypes of gastrointestinal stromal tumors and their prognostic and therapeutic implications. *Future Oncol.* **13**, 93–107 (2017). 27600498 10.2217/fon-2016-0192

[3] Bauer, S., George, S., von Mehren, M. & Heinrich, M. C. Early and Next-Generation KIT/PDGFRA Kinase Inhibitors and the Future of Treatment for Advanced Gastrointestinal Stromal Tumor. *Front. Oncol*. **11**, 672500 (2021). 10.3389/fonc.2021.672500 PMC8313277 34322383

[4] Cocco, E., Scaltriti, M. & Drilon, A. NTRK fusion-positive cancers and TRK inhibitor therapy. *Nat. Rev. Clin. Oncol.* **15**, 731–747 (2018). 30333516 10.1038/s41571-018-0113-0 PMC6419506

[5] Joensuu, H. et al. Effect of the Tyrosine Kinase Inhibitor STI571 in a Patient with a Metastatic Gastrointestinal Stromal Tumor. *N. Engl. J. Med.* **344**, 1052–1056 (2001). 11287975 10.1056/NEJM200104053441404

[6] Demetri, G. D. et al. Efficacy and safety of regorafenib for advanced gastrointestinal stromal tumours after failure of imatinib and sunitinib (GRID): an international, multicentre, randomised, placebo-controlled, phase 3 trial. *Lancet Lond. Engl.* **381**, 295–302 (2013). 10.1016/S0140-6736(12)61857-1 PMC3819942 23177515

[7] Blay, J.-Y. et al. Ripretinib in patients with advanced gastrointestinal stromal tumours (INVICTUS): a double-blind, randomised, placebo-controlled, phase 3 trial. *Lancet Oncol.* **21**, 923–934 (2020). 32511981 10.1016/S1470-2045(20)30168-6 PMC8383051

[8] Antonescu, C. R. et al. Acquired Resistance to Imatinib in Gastrointestinal Stromal Tumor Occurs Through Secondary Gene Mutation. *Clin. Cancer Res.* **11**, 4182–4190 (2005). 15930355 10.1158/1078-0432.CCR-04-2245

[9] Debiec-Rychter, M. et al. Mechanisms of resistance to imatinib mesylate in gastrointestinal stromal tumors and activity of the PKC412 inhibitor against imatinib-resistant mutants. *Gastroenterology* **128**, 270–279 (2005). 15685537 10.1053/j.gastro.2004.11.020

[10] Heinrich, M. C. et al. Molecular correlates of imatinib resistance in gastrointestinal stromal tumors. *J. Clin. Oncol. J. Am. Soc. Clin. Oncol.* **24**, 4764–4774 (2006). 10.1200/JCO.2006.06.2265 16954519

[11] Wardelmann, E. et al. Polyclonal Evolution of Multiple Secondary KIT Mutations in Gastrointestinal Stromal Tumors under Treatment with Imatinib Mesylate. *Clin. Cancer Res.* **12**, 1743–1749 (2006). 16551858 10.1158/1078-0432.CCR-05-1211

[12] Desai, J. et al. Clonal Evolution of Resistance to Imatinib in Patients with Metastatic Gastrointestinal Stromal Tumors. *Clin. Cancer Res.* **13**, 5398–5405 (2007). 17875769 10.1158/1078-0432.CCR-06-0858

[13] Miselli, F. C. et al. c-Kit/PDGFRA gene status alterations possibly related to primary imatinib resistance in gastrointestinal stromal tumors. *Clin. Cancer Res. J. Am. Assoc. Cancer Res.* **13**, 2369–2377 (2007). 10.1158/1078-0432.CCR-06-1745 17438095

[14] Heinrich, M. C. et al. Primary and secondary kinase genotypes correlate with the biological and clinical activity of sunitinib in imatinib-resistant gastrointestinal stromal tumor. *J. Clin. Oncol. J. Am. Soc. Clin. Oncol.* **26**, 5352–5359 (2008). 10.1200/JCO.2007.15.7461 PMC2651076 18955458

[15] Liegl, B. et al. Heterogeneity of kinase inhibitor resistance mechanisms in GIST. *J. Pathol.* **216**, 64–74 (2008). 18623623 10.1002/path PMC2693040

[16] Du, J. et al. Identifying Secondary Mutations in Chinese Patients with Imatinib-Resistant Gastrointestinal Stromal Tumors (GISTs) by Next Generation Sequencing (NGS). *Pathol. Oncol. Res. POR* **26**, 91–100 (2020). 31758409 10.1007/s12253-019-00770-6

[17] Li, J. et al. Clinicopathological characteristics of progressive gastrointestinal stromal tumors and heterogeneity analyses of secondary mutations. *Oncologist* **30**, oyaf110 (2025). 40377443 10.1093/oncolo/oyaf110 PMC12082821

[18] Mahadevan, D. et al. A novel tyrosine kinase switch is a mechanism of imatinib resistance in gastrointestinal stromal tumors. *Oncogene* **26**, 3909–3919 (2007). 17325667 10.1038/sj.onc.1210173

[19] Agaram, N. P. et al. Novel V600E BRAF mutations in imatinib-naive and imatinib-resistant gastrointestinal stromal tumors. *Genes. Chromosomes Cancer* **47**, 853–859 (2008). 18615679 10.1002/gcc.20589 PMC2902874

[20] Dermawan, J. K. et al. FGFR2::TACC2 fusion as a novel KIT-independent mechanism of targeted therapy failure in a multidrug-resistant gastrointestinal stromal tumor. *Genes. Chromosomes Cancer* **61**, 412–419 (2022). 35170141 10.1002/gcc.23030 PMC9194600

[21] Machado, I. et al. ETV6::NTRK3 Fusion-Positive Wild-Type Gastrointestinal Stromal Tumor (GIST) with Abundant Lymphoid Infiltration (TILs and Tertiary Lymphoid Structures): A Report on a New Case with Therapeutic Implications and a Literature Review. *Int. J. Mol. Sci.* **25**, 3707 (2024). 38612518 10.3390/ijms25073707 PMC11011305

[22] Ranjbarian, T. et al. A Systematic Review with a Demonstrative Case of KIT and DOG-1 Expressing Gastrointestinal Stromal Tumors Harboring ETV6–NTRK3 Fusions. *Clin. Cancer Res.* **31**, 2056–2061 (2025). 39992648 10.1158/1078-0432.CCR-24-3203 PMC12081180

[23] Soda, M. et al. Identification of the transforming EML4–ALK fusion gene in non-small-cell lung cancer. *Nature* **448**, 561–566 (2007). 17625570 10.1038/nature05945

[24] Tang, H. et al. Fusion genes in cancers: Biogenesis, functions, and therapeutic implications. *Genes Dis.* **12**, 101536 (2025). 40584293 10.1016/j.gendis.2025.101536 PMC12205800

[25] Taguchi, T. et al. Conventional and molecular cytogenetic characterization of a new human cell line, GIST-T1, established from gastrointestinal stromal tumor. *Lab. Investig. J. Tech. Methods Pathol.* **82**, 663–665 (2002). 10.1038/labinvest.3780461 12004007

[26] Brenca, M. et al. Transcriptome sequencing identifies ETV6–NTRK3 as a gene fusion involved in GIST. *J. Pathol.* **238**, 543–549 (2016). 26606880 10.1002/path.4677

[27] Atiq, M. A. et al. Mesenchymal tumors of the gastrointestinal tract with NTRK rearrangements: a clinicopathological, immunophenotypic, and molecular study of eight cases, emphasizing their distinction from gastrointestinal stromal tumor (GIST). *Mod. Pathol.* **34**, 95–103 (2021). 32669612 10.1038/s41379-020-0623-z

[28] Antonescu, C. R. et al. Dedifferentiation in gastrointestinal stromal tumor to an anaplastic KIT-negative phenotype: a diagnostic pitfall: morphologic and molecular characterization of 8 cases occurring either de novo or after imatinib therapy. *Am. J. Surg. Pathol.* **37**, 385–392 (2013). 23348204 10.1097/PAS.0b013e31826c1761 PMC3728887

[29] Choi, J. J., Sinada-Bottros, L., Maker, A. V. & Weisenberg, E. Dedifferentiated gastrointestinal stromal tumor arising de novo from the small intestine. *Pathol. Res. Pract.* **210**, 264–266 (2014). 24484970 10.1016/j.prp.2013.12.008

[30] Karakas, C., Christensen, P., Baek, D., Jung, M. & Ro, J. Y. Dedifferentiated gastrointestinal stromal tumor: Recent advances. *Ann. Diagn. Pathol.* **39**, 118–124 (2019). 30661742 10.1016/j.anndiagpath.2018.12.005

[31] Malik, F. et al. Dedifferentiation in SDH-Deficient Gastrointestinal Stromal Tumor: A Report With Histologic, Immunophenotypic, and Molecular Characterization. *Pediatr. Dev. Pathol. J. Soc. Pediatr. Pathol. Paediatr. Pathol. Soc.* **22**, 492–498 (2019). 10.1177/1093526619846222 31072206

[32] Gasparotto, D. et al. Quadruple-Negative GIST Is a Sentinel for Unrecognized Neurofibromatosis Type 1 Syndrome. *Clin. Cancer Res. J. Am. Assoc. Cancer Res.* **23**, 273–282 (2017). 10.1158/1078-0432.CCR-16-0152 27390349

[33] Sigalotti, L. et al. Proximal and Classic Epithelioid Sarcomas are Distinct Molecular Entities Defined by MYC/GATA3 and SOX17/Endothelial Markers, Respectively. *Mod. Pathol. J. U. S. Can. Acad. Pathol. Inc.* **38**, 100647 (2025). 10.1016/j.modpat.2024.100647 39491746

[34] Dobin, A. et al. STAR: ultrafast universal RNA-seq aligner. *Bioinforma. Oxf. Engl.* **29**, 15–21 (2013). 10.1093/bioinformatics/bts635 PMC3530905 23104886

[35] Li, B. & Dewey, C. N. RSEM: accurate transcript quantification from RNA-Seq data with or without a reference genome. *BMC Bioinforma.* **12**, 323 (2011). 10.1186/1471-2105-12-323 PMC3163565 21816040

[36] Uhrig, S. et al. Accurate and efficient detection of gene fusions from RNA sequencing data. *Genome Res* **31**, 448–460 (2021). 33441414 10.1101/gr.257246.119 PMC7919457

[37] Behera, S. et al. Comprehensive genome analysis and variant detection at scale using DRAGEN. *Nat. Biotechnol.* **43**, 1177–1191 (2025). 39455800 10.1038/s41587-024-02382-1 PMC12022141

[38] Franken, N. A. P., Rodermond, H. M., Stap, J., Haveman, J. & van Bree, C. Clonogenic assay of cells in vitro. *Nat. Protoc.* **1**, 2315–2319 (2006). 17406473 10.1038/nprot.2006.339

