# Supplementary material for: KIT Mutation-NTRK fusion oncogenic driver switch: a novel mechanism of acquired imatinib resistance in GIST
Source: NPJ Precis Oncol. 2026 Jan 21;10:75. doi: 10.1038/s41698-026-01289-1 (PMC12923657; doi:10.1038/s41698-026-01289-1)
Supplement: Supplementary file 1 — Supplementary Table [file 41698_2026_1289_MOESM1_ESM.pdf]

Supplementary Table 1. cDNA and protein sequence of the EML4::NTRK3 chimera

> EML4::NTRK3 fusion cDNA (EcoRI-EcoRI cloning)

GAATTCGAGCTCGGTACCC
ATGGACGGTTTCGCCGGCAGTCTCGATGATAGTATTTCTGCTGCAAGTACTTCTGATGTTCAAGATCGCCTGTCAGCTCTTGAGT
CACGAGTTCAGCAACAAGAAGATGAAATCACTGTGCTAAAGGCGGCTTTGGCTGATGTTTTGAGGCGTCTTGCAATCTCTGAAG
ATCATGTGGCCTCAGTGAAAAAATCAGTCTCAAGTAAAGGTCCCGTGGCTGTCATCAGTGGTGAGGAGGACTCAGCCAGCCCAC
TGCACCACATCAACCACGGCATCACCACGCCCTCGTCACTGGATGCGGGGCCCGACACTGTGGTCATTGGCATGACTCGCATC
CCTGTCATTGAGAACCCCCAGTACTTCCGTCAGGGACACAACTGCCACAAGCCGGACACGTATGTGCAGCACATTAAGAGGAG
AGACATCGTGCTGAAGCGAGAACTGGGTGAGGGAGCCTTTGGAAAGGTCTTCCTGGCCGAGTGCTACAACCTCAGCCCGACCA
AGGACAAGATGCTTGTGGCTGTGAAGGCCCTGAAGGATCCCACCCTGGCTGCCCGGAAGGATTTCCAGAGGGAGGCCGAGCT
GCTCACCAACCTGCAGCATGAGCACATTGTCAAGTTCTATGGAGTGTGCGGCGATGGGGACCCCCTCATCATGGTCTTTGAATA
CATGAAGCATGGAGACCTGAATAAGTTCCTCAGGGCCCATGGGCCAGATGCAATGATCCTTGTGGATGGACAGCCACGCCAGG
CCAAGGGTGAGCTGGGGCTCTCCCAAATGCTCCACATTGCCAGTCAGATCGCCTCGGGTATGGTGTACCTGGCCTCCCAGCAC
TTTGTGCACCGAGACCTGGCCACCAGGAACTGCCTGGTTGGAGCGAATCTGCTAGTGAAGATTGGGGACTTCGGCATGTCCAG
AGATGTCTACAGCACGGATTATTACAGGGTGGGAGGACACACCATGCTCCCCATTCGCTGGATGCCTCCTGAAAGCATCATGTA
CCGGAAGTTCACTACAGAGAGTGATGTATGGAGCTTCGGGGTGATCCTCTGGGAGATCTTCACCTATGGAAAGCAGCCATGGTT
CCAACTCTCAAACACGGAGGTCATTGAGTGCATTACCCAAGGTCGTGTTTTGGAGCGGCCCCGAGTCTGCCCCAAAGAGGTGT
ACGATGTCATGCTGGGGTGCTGGCAGAGGGAACCACAGCAGCGGTTGAACATCAAGGAGATCTACAAAATCCTCCATGCTTTG
GGGAAGGCCACCCCAATCTACCTGGACATTCTTGGCTAG
TGGTGGCTGGTGGTCATGAATTC

>EML4::NTRK3 fusion protein

MDGFAGSLDDSISAASTSDVQDRLSALESRVQQQEDEITVLKAALADVLRRLAISEDHVASVKKSVSSKGPVAVISGEEDSASPLHHIN
HGITTPSSLDAGPDTVVIGMTRIPVIENPQYFRQGHNCHKPDTYVQHIKRRDIVLKRELGEGAFGKVFLAECYNLSPTKDKMLVAVKAL
KDPTLAARKDFQREAELLTNLQHEHIVKFYGVCGDGDPLIMVFEYMKHGDLNKFLRAHGPDAMILVDGQPRQAKGELGLSQMLHIAS
QIASGMVYLASQHFVHRDLATRNCLVGANLLVKIGDFGMSRDVYSTDYYRVGGHTMLPIRWMPPESIMYRKFTTESDVWSFGVILWEI
FTYGKQPWFQLSNTEVIECITQGRVLERPRVCPKEVYDVMLGCWQREPQQRLNIKEIYKILHALGKATPIYLDILG

Color code: Black, pLPC vector; Blue, *EML4;* Green, *NTRK3*

Supplementary Table 2. STR profile of GIST-T1 cell line

KIT exon 11 heterozygous mutation p.Val560_Tyr578del (c.1679_1735del)

MARKER	***STR***
AMEL	*X*
D8S1179	*13;13*
D21S11	*29;31*
D7S820	*11;12*
CSF1PO	*10;12*
D3S1358	*15;15*
TH01	*7;9*
D13S317	*11;11*
D16S539	*11,13*
D2S1338	*17;26*
D19S433	*14;15*
vWA	*14;14*
TPOX	*11;11*
D18S51	*14;14*
D5S818	*10;10*
FGA	*22;23*

Supplementary Figure S1

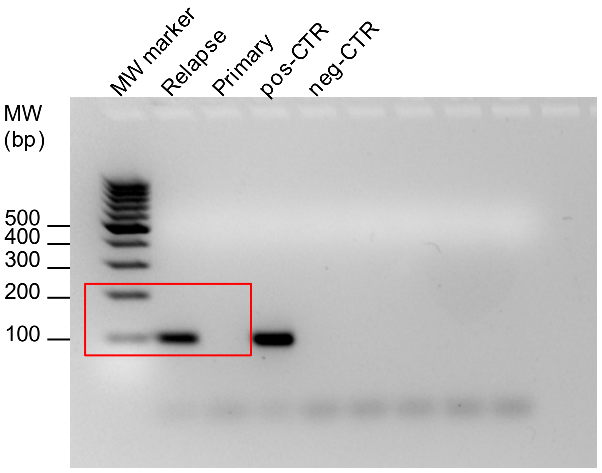

Uncropped original gel corresponding to the panel shown in Figure 3b. The pLPC-E4N3 plasmid was used as a positive control (pos-CTR), while the no-template reaction served as a negative control (neg-CTR). The lanes included in the figure are highlighted with a red rectangle.

Supplementary Figure S2

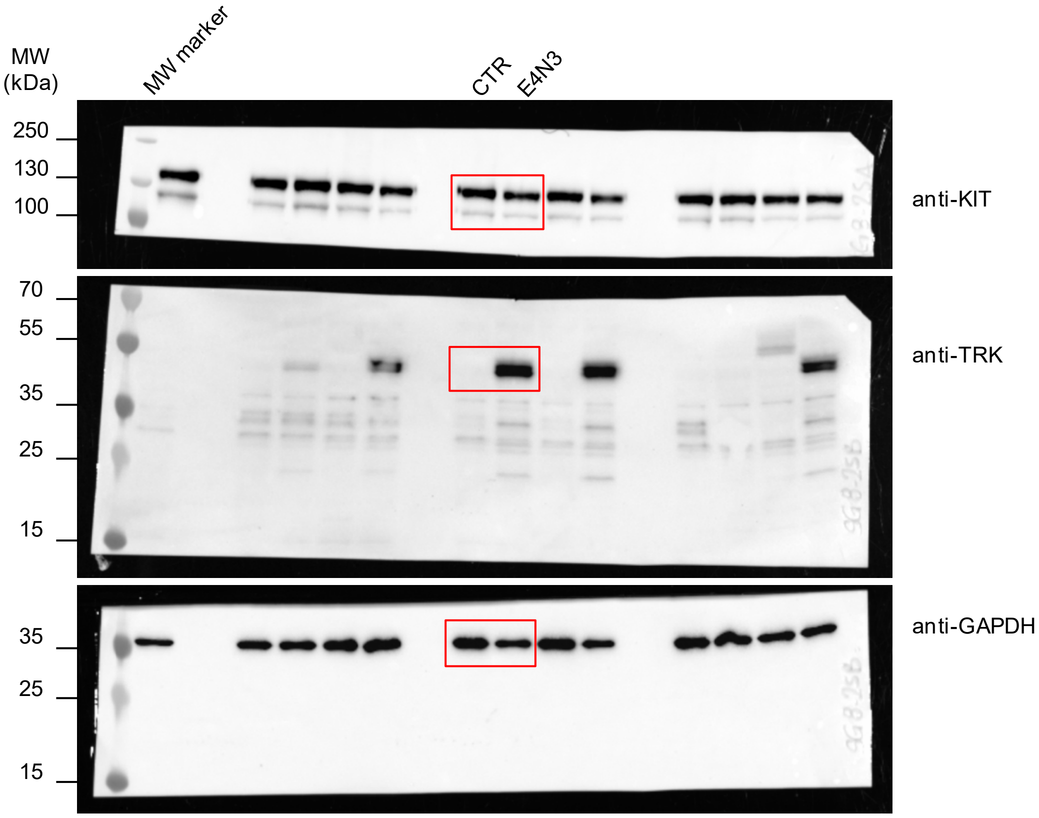

Uncropped original Western blots for KIT, TRK, and GAPDH (loading control), corresponding to the panels shown in Figure 3f. The two lanes included in the figure are highlighted with red rectangles.
